# Global health education: a pilot in trans-disciplinary, digital instruction

**DOI:** 10.3402/gha.v6i0.20747

**Published:** 2013-05-02

**Authors:** Heather Wipfli, David J. Press, Virginia Kuhn

**Affiliations:** 1 USC Institute for Global Health, University of Southern California, Los Angeles, CA, USA; 2 USC Department of Preventive Medicine, University of Southern California, Los Angeles, CA, USA; 3 Global Health Program, Association of Pacific Rim Universities, Singapore; 4 School of International Relations, USC Dornsife College of Letters, Arts and Sciences, University of Southern California, Los Angeles, CA, USA; 5 USC School of Cinematic Arts, USC Institute for Multimedia Literacy, University of Southern California, Los Angeles, CA, USA

**Keywords:** academic programs, digital media, education, global health, multimedia learning, technology

## Abstract

**Background:**

The development of new global health academic programs provides unique opportunities to create innovative educational approaches within and across universities. Recent evidence suggests that digital media technologies may provide feasible and cost-effective alternatives to traditional classroom instruction; yet, many emerging global health academic programs lag behind in the utilization of modern technologies.

**Objective:**

We created an inter-departmental University of Southern California (USC) collaboration to develop and implement a course focused on digital media and global health.

**Design:**

Course curriculum was based on core tenants of modern education: multi-disciplinary, technologically advanced, learner-centered, and professional application of knowledge. Student and university evaluations were reviewed to qualitatively assess course satisfaction and educational outcomes.

**Results:**

‘New Media for Global Health’ ran for 18 weeks in the Spring 2012 semester with N=41 students (56.1% global health and 43.9% digital studies students). The course resulted in a number of high quality global health-related digital media products available at http://iml420.wordpress.com/. Challenges confronted at USC included administrative challenges related to co-teaching and frustration from students conditioned to a rigid system of teacher-led learning within a specific discipline. Quantitative and qualitative course evaluations reflected positive feedback for the course instructors and mixed reviews for the organization of the course.

**Conclusion:**

The development of innovative educational programs in global health requires on-going experimentation and information sharing across departments and universities. Digital media technologies may have implications for future efforts to improve global health education.

Precipitous technological advances and resulting cultural shifts have challenged traditional university pedagogy – defined by in-class transfer of information from instructor to student and disciplinary distinctiveness. These trends have broad implications for public health and medical education (1–5). In order to spark reforms in health professional education, a recent *Lancet* commission called for a number of transformations, including increased cross-disciplinary collaboration and improved integration of technology (6). The responses from health professionals, university officials, and students have invariably underscored the complexities inherent in the tasks ahead (7).

Global health is a booming new field of study that investigates the interplay between medical, economic, and environmental factors as they impact human health. The number of universities with a program or institution with global health in its name rose from just one in 1999 to over 40 in 2009 (8). Since global health is a dynamic, multi-disciplinary field practiced in a highly networked world, emerging global health programs provide a unique opportunity to cultivate and apply innovative educational approaches within the health sciences (9–18). Recent reports from global health academic programs in the United States have reported a variety of benefits and challenges associated with increased investments in innovative educational approaches (19–21). Yet, evidence regarding the effectiveness of efforts to integrate digital media technologies into global health classrooms setting remains lacking; thus, global health academic programs continue to be largely structured and delivered within traditional frameworks (8). This deficit should be of particular concern to global health educators, given that organizations increasingly: (a) leverage digital public space; (b) require multimedia submissions for grant applications (www.grandchallenges.org); and (c) require sophisticated technological knowledge for the entering workforce.

The University of Southern California (USC) is an example of a university that has recently invested in global health infrastructure and academic programs. USC offers a broad range of curricula in global health, including a Bachelor of Science (BS) in Global Health, a Minor in Global Health, a Masters in Public Health (MPH) with a global health concentration, and a Master of Science (MS) in Global Medicine. The BS in Global Health is composed of a spectrum of courses in the basic sciences, international relations, and health promotion disease prevention. The majority of global health classes at USC remain disciplinarily distinct and instructor-driven, with limited utilization of technology to enhance the learning experience. To address this weakness, USC faculty in global health and at the Institute for Multimedia Literacy (IML) collaborated to develop a multi-disciplinary course that would integrate new technologies into global health coursework to increase fluency in the application of technologies for global health objectives.

Digital studies (or new media studies) attends to the close interrelationships among technology, culture, communication, and expression across the registers of text, audio, video, and interactivity to impact large-scale multimedia practices (22–26). Digital studies apply systems thinking to the service of real-world issues but may suffer from a lack of educational models and systematic approaches for applying discipline-specific knowledge to real-world problems (27–29). Consequently, enhanced linkages between global health and digital studies may improve the academic experience and educational outcomes for students in each.

This article highlights benefits and challenges identified throughout our pilot collaborative course, with particular emphasis on those aspects that carry wide implications for global health pedagogy.

## Present investigation

### Design

The instructor from the USC Institute for Global Health (HW) is an expert in international policy; the instructor from the USC IML (VK) is an expert in new media studies. Course concept arose when instructors met through a university-wide program aimed at harnessing the expertise of USC for global health. Our course design was competency-driven, with the ultimate goal of fostering digital media learning outcomes among global health students from the Minor and BS programs in global health and enhancing research skills among digital studies students from the Minor in digital studies. We developed projects to build these competencies (Table 1). Students were to work in diverse teams and present their work in novel ways that allowed connections to appear that were not readily apparent in traditional representations. The faculty spent extensive time identifying competencies, developing the syllabus, and preparing weekly seminars. Weekly seminars would provide a forum to share, reflect upon, and collaborate on projects. Online course management would be implemented through a personalized wiki platform for students to access course information, post assignments, carry out peer review, and share resources. The class would utilize the production facilities and Mac-based computer labs at the USC IML. Curricula choices were made collaboratively in ways that would enhance the course goals for each discipline without compromising either. With regard to the specific digital media software platforms selected for use in the course, the emphasis was on how to learn to work with platforms rather than to become experts in any one specific tool.

**Table 1:** Course competencies in digital media for global health students^a^

Theme	Competencies
Partnership and teamwork	(1) Understand the importance of promoting teams with varied backgrounds and expertise in global health. (2) Function effectively as a member of a diverse multi-disciplinary team.
Sociocultural and political determinants	(1) Ability to develop and visualize stakeholder maps in support of global health policy and programs. (2) Be able to describe and visualize differences in national health systems and their influence on transnational health issues.
Strategic analysis	(1) Be able to interpret and visualize metrics used to characterize global health problems, including mortality and incidence rates, prevalence, and disease burden. (2) Present data in multiple forms to assist in identifying how demographic and other factors shape health in a specified community, country, or region. (3) Identify appropriate digital tools and resources for application to global health problems.
Capacity strengthening	(1) Assess and address the capacity of partners and collaborators in regards to digital media. (2) Understand the importance of communicating information about local capacity as a means to promoting sustainability.
Project management	(1) Design context-appropriate digital resources based on scientific evidence in consultation with stakeholders. (2) Develop digital resources to secure donor funding and stakeholder support. (3) Utilize digital tools for program monitoring and evaluation.
Health equity, human rights, and social justice	(1) Apply social justice and human rights concepts and methods to the design, implementation, and evaluation of new media pieces about global health topics and themes.
Ethical reasoning and professional practice	(1) Analyze ethical and professional issues that arise in conducting and communicating global health work, including in response to public health emergencies. (2) Produce digital media that upholds public health practice standards.

a Categories based on the American School of Public Health Global Health Competency Model. (Association of Schools of Public Health. 2011. Global Health Competency Model. Retrieved from http://www.asph.org/document.cfm?page=1084; cited 15 January 2012.)

The course was divided into three modules, each one lasting roughly 5 weeks and resulting in distinct digital projects (Table 2). The first module focused on global disease surveillance and data visualization. Lectures focused on trends in the global disease burden and social determinants of disease in addition to the role of color, graphics, and text in the expression of knowledge. Students were introduced to a range of online data visualization tools, including Many Eyes (30), Dipity (31), Wordle (32), Gliffy (33), and Omnigraffle (34). For their project, students independently researched disease trends and selected a single data set from an online data repository, including from the United Nations, Centers for Disease Control and Prevention, World Health Organization, Gap Minder, and Google Public Data. Students explored four distinct ways to visualize their data using different platforms and tools and then described the ways in which their visualization shifted the meaning of the data depending on the context and presentation. This project was done individually and, in order to provide further scaffolding, students were required to submit their data set and project plan for approval before completing the project.

**Table 2:** Course design

Course title	Digital media for global health
Credits/time	4 credits; One 3.5-hour weekly seminar over 18 weeks
Modules	(1) Visualizing shifts in global burden of morbidity and mortality. (2) Digitally mapping the epidemiology of disease. (3) Digital argument and the HIV epidemic.
Grading	(1) Visualization of disease burden project (25%). (2) Geo-mapping the Haitian Cholera Outbreak Project (25%). (3) Digital argument project on HIV+the law (web-based documentary) (25%) (4) Reading responses and peer review (25%).
Required texts	(1) (A. G. Johnson, 2005; S. Johnson, 2006; Nolen, 2008). (2) Weekly journal articles.

The second section of the course focused on the spread of infectious disease and geospatial mapping. The unit began with a reading of Steven Johnson's *The Ghost* Map – a historical account of how John Snow tracked the source of the infamous 1,854 cholera outbreak in London (35). We emphasized Snow as a systems-level thinker, drawing connections across anthropology, urban planning, and the life sciences, while encouraging students to consider the role of mapping in providing context and visibility to these transversal relationships. Students read media pieces and peer-reviewed articles related to the 2012 cholera outbreak in Haiti and developed an interactive geo-mapping assignment as a way to connect past with present using Hypercities (36), a digital platform developed by academic researchers. Students were placed in four teams equally composed of digital studies and global health students to research four determinants of the Haitian cholera outbreak – population characteristics, national infrastructure, the health system, and international aid. After collecting data, narratives, videos, and images on their respective topic, the teams curated resources and posted them as a self-contained ‘layer’ of data on an online geospatial mapping platform (http://hypercities.ats.ucla.edu).

The third module focused on human immunodeficiency virus (HIV) and the law. Small groups of three or four students produced short films using words, images (still and moving), and animation to create a nuanced academic argument. Nolen's *28: Stories on Aids in Africa* (37), policy briefs, and peer-reviewed articles served as source material, in addition to a guest lecture from a legal expert in the field and video testimony from the Commission on HIV and the Law (38). Concurrently, students received training in how to find, capture, and convert the existing video assets online [SnapZ (39), Reelsurfer (40), Zamzar (41)] in addition to training in video editing [Final Cut Pro (42)] and posting to the web [Quicktime (43)]. Digital studies students, being well versed in these tools, were given tutorials in special effects in order to help animate dynamic visualizations to enhance the testimonial video.

Students were evaluated based on classroom participation, project execution, weekly reading responses, and peer review. Students were expected to evidence their ability to critically evaluate the linkages between theory and practice in global health and digital studies (Table 3). Students, in turn, had four opportunities to evaluate the course and instructors: (1) after the first project was graded; (2) through a reflective essay following the second project; (3) through video statements after the third project; and (4) through formal University course evaluations on the final day of the course. Selected students were also interviewed on camera during the summer after the class. Instructors met after each evaluation to discuss the feedback, to undertake mid-course corrections, and to inform future versions of the course.

**Table 3:** Digital project grading rubric

Conceptual core	Research component
A project's controlling idea must be apparent, productively aligned with one or more multimedia genres, and effectively engage with the primary issue/s of the subject area into which it is intervening.	Each project must display evidence of substantive research and thoughtful engagement with its subject matter, use a variety of credible sources appropriately cited, and optimally deploy more than one approach to an issue.
Form+content	Creative realization
The project's structural or formal elements must serve the conceptual core; design decisions must be deliberate, controlled, and defensible; and efficacy should be unencumbered by technical problems.	The project must approach the subject in a creative or innovative manner using media and design principles effectively and must achieve significant goals that could not be realized on paper alone.

Quantitative course evaluations from Spring 2011 were compared to the same 12 measures collected in Spring 2012, using a Likert scale from 1=poor to 5=excellent. Open-ended course evaluations were collected at the same time to qualitatively assess student feedback. Qualitative data were coded according to general thematic areas in order to demarcate informative segments in the data.

### Results

In Spring 2012, two four-unit courses, ‘New Media for Social Change’ (listed through the IML at the USC School of Cinematic Arts), and ‘Case Studies in Global Health’ (listed through the Keck School of Medicine of USC), were convened concurrently. The combined ‘New Media for Global Health’ course ran for 18 weeks with 18 digital studies and 23 global health students. Students developed a number of high quality digital media products displayed at the annual IML Showcase, which were subsequently made available online (http://iml420.wordpress.com/).

Attendance was consistently high, due in part to the use of class time for project work and hands-on instruction in media tools. In addition, students spent substantial time working on assignments outside of class. Most project content was well researched and referenced, and presented in innovative ways. Grades for most global health students improved throughout the semester, with the lowest scores received on the first module and the highest scores on the final module. Student evaluations by professors demonstrated substantial improvements in baseline competencies and knowledge among global health and digital studies students. The largest competency gains were observed for strategic analysis and project management. The emergent and complex technology of the Hypercities platform (36) used for the second project provided the greatest technological challenge to both global health and digital studies students.

Global health student feedback on the first project focused on ‘unclear instructions’ and ‘unfair expectations.’ Following the second project, global health students highlighted their frustration with the division of labor within their inter-disciplinary teams, noting that they failed to learn about the digital platform since digital studies students dominated this aspect of the project. This frustration was mitigated through in class discussions, as evidenced by evaluations following the third project, which indicated a more equal division of labor. Some global health students enjoyed the project-based aspect of the class, one remarking: ‘I loved doing projects instead of writing essays’; and ‘This was definitely a lot more interesting than just studying case studies alone.’

Quantitative analyses indicated non-significant reductions in satisfaction between the case studies approach in 2011 and the 2012 course. On a Likert scale of 1–5, median scores in overall satisfaction dropped by 0.5 (standard deviation, 1.58) for the instructor and 0.7 (standard deviation, 1.59) for the course. For the Spring 2012 semester, the highest score was for the measure: ‘[Instructor] was enthusiastic about communicating the subject matter’; the lowest score was for the measure: ‘[Instructor] organized course to achieve those goals.’ Qualitative analyses on the 11 open-ended course evaluations demonstrated substantial approval of the course instructor and mixed reviews of the course. Approximately 90.9% of students provided responses expressing the thematic area that the instructor was knowledgeable, intelligent, had experience in global health, and/or was adept at explaining subject matter. The majority of students (63.6%) expressed negative qualitative feedback in the thematic areas of course organization, integration, execution, and unclear expectations.

Final student statements on the class are available at http://vimeo.com/47376843. These statements provide a more nuanced perspective than what was collected during the course evaluations. A convenience sample of excerpts from the final student statements is provided in Table 4.

**Table 4:** Selected student feedback by thematic area

Interdisciplinary faculty collaboration and balance	‘At first, I was worried that I would be unable to perform well due to my inexperience in digital media, and I thought it would be unfair for global health students. However, after the completion of the semester, I was surprised to see how integrated digital media is to global health and the idea of having the [digital studies] and [global health] students work together was ingenious.’
Assessing student performance	‘I really like how the class does not focus on exams and grades so much, which allows me less restraints, but unfortunately, when it comes down to it, this class will be affecting my GPA, and I would like to know how to perform under your standards.’
Value of student experience	‘I remember first hesitantly stepping into the classroom, or more accurately put, a theater, and being completely bewildered as to what I got myself into not realizing that I'll have one of the most valuable experiences I've ever had at SC thus far.’
	‘This was a real life experience.’

## Discussion

This experimental course provided a model for global health education using digital media technologies. The course brought together faculty and students in digital studies and global health to explore systematic complexities underlying both fields to create digitally rendered resources in the service of real-world problems. There were a number of successes, including the dynamic interaction between students and the high-quality media projects produced.

Our results contribute to the growing body of evidence supporting innovative usage of electronic media in the classroom setting. For example, the Hopkins Center for Clinical Global Health Education has reported that educational programs utilizing multiple information technology tools to teach and learn have been feasible and cost-effective (20).

There were a number of challenges, including the compounded organizational difficulties, related to co-teaching a trans-disciplinary course for the first time and meeting the expectations of students who have been conditioned to a rigid system of disciplinarily-distinct and teacher-led learning. One challenge was determining the right ‘balance’ between global health and digital studies content to ensure that the needs of both cohorts were met. Eventually both cohorts complained that the other discipline dominate the course. In addition, the instructors were at times unfamiliar with the jargon of their counterpart's discipline, which resulted in active conversations between the instructors in front of the class. For example, the word ‘surveillance’ has very distinct connotations for global health and media professionals. We anticipated these interactions, as they often arise in collaborative research projects. Many students, however, expressed discomfort from these exchanges, as expressed in their feedback: ‘… the professors were not always on the same page’; ‘I felt like I was listening to them talk to each other and not learning anything.’ In our opinion, exposure to this inter-disciplinary exchange offered a valuable learning experience to students and faculty alike.

Student assessment was a challenge. The faculty attempted to provide clear guidelines for each assignment – detailed instruction sheets with evaluation criteria linked to the course wiki were reviewed in class. Assessment criteria emphasized the importance of content, form, and creativity. A certain level of complexity in assessment may be acceptable in higher education, particularly for trans-disciplinary courses aimed at competency building. However, this does not fit well with student expectations, particularly among pre-medical students whose perceived needs for an ‘A’ may create discomfort with non-traditional deliverables and assessment rubrics. To mitigate student anxiety in grading, we routinely assured students that the faculty-lead from their respective field would be ultimately responsible for grading their work based primarily on competencies in their respective discipline. Regardless, students complained that they were not ‘learning what I signed up for’, with one stating, ‘At times I was driven completely out of my element and unable to exercise my strengths.’ Plans are in place to offer the course again, implementing the knowledge and recommended changes gleaned from the pilot, including more advanced notification of the trans-disciplinary nature of the course in the course description.

## Conclusion

The development of successful training programs for global health professionals will require on-going experimentation and information sharing between programs. It requires extraordinary effort from faculty not traditionally awarded for such work and flexibility on the part of students who may be risk averse. ‘New Media for Global Health’ provided students with an applied learning experience similar to what they may face after graduation. This was achieved by creating trans-disciplinary student teams that were equipped to develop projects for public dissemination. The course also led students into unfamiliar territory – similar to the feeling that many will experience when working in the cross-cultural and cross-disciplinary field of global health. We hope that others engaged in public heath educational efforts will share their experiences so that we may learn together.
